# A New Tumor Burden Score and Albumin–Bilirubin Grade-Based Prognostic Model for Hepatocellular Carcinoma

**DOI:** 10.3390/cancers14030649

**Published:** 2022-01-27

**Authors:** Shu-Yein Ho, Po-Hong Liu, Chia-Yang Hsu, Yi-Hsiang Huang, Jia-I Liao, Chien-Wei Su, Ming-Chih Hou, Teh-Ia Huo

**Affiliations:** 1 Division of Gastroenterology and Hepatology, Min-Sheng General Hospital, Taoyuan 330, Taiwan; zawzaw222@gmail.com; 2 Department of Medical Research, Taipei Veterans General Hospital, Taipei 11217, Taiwan; 3 School of Medicine, National Yang Ming Chiao Tung University, Taipei 11217, Taiwan; stuartliu@gmail.com (P.-H.L.); chiayanghsu2@gmail.com (C.-Y.H.); yhhuang@vghtpe.gov.tw (Y.-H.H.); alohawhs@hotmail.com (J.-I.L.); cwsu2@vghtpe.gov.tw (C.-W.S.); mchou@vghtpe.gov.tw (M.-C.H.); 4 Department of Internal Medicine, University of Texas Southwestern Medical Center, Dallas, TX 75390, USA; 5 Veterans Affairs Sierra Nevada Healthcare System, Reno, NV 89502, USA; 6 Division of Gastroenterology and Hepatology, Department of Medicine, Taipei Veterans General Hospital, Taipei 11217, Taiwan; 7 Institute of Clinical Medicine, National Yang Ming Chiao Tung University, Taipei 11217, Taiwan; 8 Institute of Pharmacology, National Yang Ming Chiao Tung University, Taipei 11217, Taiwan

**Keywords:** tumor burden score, albumin–bilirubin grade, hepatocellular carcinoma, prognosis

## Abstract

**Simple Summary:**

The survival of patients with hepatocellular carcinoma (HCC) is highly variables, due to heterogeneous tumor burden and liver dysfunction. Tumor burden score (TBS) is a continuous variable to measure the extent of tumor involvement, and the albumin–bilirubin (ALBI) grade is an objective model to estimate hepatic functional reserve. Six prognostic predictors—including TBS, ALBI grade, ascites, serum α-fetoprotein level, vascular invasion or distant metastasis, and performance status—were linked with survival in a multivariate Cox model. We used these predictors to establish a new prognostic model—the TBS–ALBI system—to predict patient outcomes. Significant survival differences were found in different TBS–ALBI scores in the derivation and validation cohorts. This new system can also discriminate survival differences in patients with different viral etiologies, cancer stages, and treatment modalities. This study shows that the TBS–ALBI system is a feasible and user-friendly prognostic model for HCC.

**Abstract:**

The prognosis of hepatocellular carcinoma (HCC) varies widely due to variable tumor extent and liver reserve. We aimed to develop and validate a new prognostic model based on tumor burden score (TBS) and albumin–bilirubin (ALBI) grade for HCC. We prospectively identified 3794 HCC patients who were randomized into derivation and validation groups. Survival predictors were evaluated by a multivariate Cox model. The TBS–ALBI system allocated two points for high TBS and ALBI grade 3, and one point each for the presence of ascites, serum α-fetoprotein ≥ 400 ng/mL, vascular invasion or distant metastasis, performance status 2–4, medium TBS, and ALBI grade 2, with a maximal score of 8 points. Significant survival differences were found across different TBS–ALBI score groups in the validation cohort (all *p* < 0.001). The TBS–ALBI system had the lowest corrected Akaike information criterion (AICc) and the highest homogeneity compared with other proposed staging models. The discriminative ability of the TBS–ALBI system was consistently stable across different viral etiologies, cancer stages, and treatment strategies. Conclusions: This new TBS–ALBI system is a feasible and robust prognostic system in comparison with other systems; it is a user-friendly tool for long-term outcome assessment independent of treatment modality and cancer stage in HCC.

## 1. Introduction

Hepatocellular carcinoma (HCC) is a highly prevalent liver cancer, and was the fourth most common cause of cancer-related death in 2018 globally [1]. The major etiologies of HCC are chronic hepatitis B and C virus (HBV, HCV) infection, alcoholism, and nonalcoholic fatty liver disease (NAFLD) [2]. Surgery, liver transplant, and local ablation are primarily indicated for early-stage HCC [2,3]. For those belonging with intermediate and advanced disease, transarterial chemoembolization (TACE) and systemic therapies, such as targeted therapy or immunotherapy, are usually recommended [4,5].

Cancer staging plays a crucial role in the management of HCC [6]. The Barcelona Clinic Liver Cancer (BCLC) system has been widely used for prognostic prediction and treatment guidance, and is endorsed by current HCC practice guidelines [2,3]. The reported survival predictors include tumor burden, severity of liver dysfunction, and performance status. Notably, tumor diameter and nodules indicate the extent of the tumor in HCC, and are included in the BCLC and Hong Kong Liver Cancer (HKLC) staging systems. These two parameters act in a dichotomous fashion with arbitrary cutoffs. The Milan criteria and the Up-to-7 criteria have also been used to evaluate the extent of tumors in HCC [7,8]. Still, these two models have limitations due to their categorical allocation, and it could be quite difficult to evaluate the prognosis for patients with variable tumor size and nodules. Investigators suggested the use of continuous tumor diameter and nodules to represent tumor burden. Mazzaferro et al. proposed the concept of a “metro-ticket model”: an increase in the diameter of the tumor and/or number of lesions and the decrease in patient survival [9]. Alternatively, continuous variables—such as total tumor diameter (TTD) and total tumor volume (TTV)—have been suggested to assess tumor extent in HCC, but these two models still harbor inevitable shortcomings [10,11]. Recently, Sasaki et al. proposed the tumor burden score (TBS), which includes tumor diameter and number of nodules as continuous variables to estimate outcomes in colorectal liver metastasis [12]. TBS was later applied to discriminate survival in HCC patients receiving partial hepatectomy and liver transplant [13,14]. The Italian Liver Cancer (ITA.LI.CA) group also used TBS to stratify prognosis in HCC patients undergoing different curative and non-curative modalities [15], indicating that TBS could be a reliable surrogate indicator of tumor burden.

The severity of liver injury is an important predictor in treating HCC. The Child–Turcotte–Pugh (CTP) classification is used to indicate the severity of cirrhosis in the BCLC, HKLC, Cancer of the Liver Italian Program (CLIP), Taipei Integrated Scoring System (TIS), and Japan Integrated System (JIS) staging systems. However, the CTP classification has potential drawbacks, owing to its subjective variables with arbitrarily defined cutoffs [16]. The model for end-stage liver disease (MELD) score has also been used to assess liver injury [17]. More recently, the albumin–bilirubin (ALBI) grade—a simple and objective method based solely on serum levels of albumin and bilirubin—was proposed to evaluate liver dysfunction, and has been validated by several research groups [18,19,20,21,22]. Although many HCC staging systems—including BCLC, HKLC, TIS, JIS, CLIP, Okuda, Tokyo, and tumor–node–metastasis (TNM)—have been used to predict the outcome of HCC [6], the optimal staging system remains unclear. In this study, we aimed to establish a new prognostic model based on TBS and ALBI grade (the TBS–ALBI system) for HCC, and its performance was comprehensively compared with other currently used staging systems.

## 2. Materials and Methods

### 2.1. Patient Characteristics

Between 2002 and 2017, we prospectively identified 3794 HCC patients in Taipei Veterans General Hospital, who were retrospectively analyzed in this study. At diagnosis, patients’ demographic data, tumor extent, severity of liver dysfunction, tumor staging, and treatments were recorded; their survival status was inspected every 3–4 months during the disease course until death or cessation of follow-up. Patients with a confirmed diagnosis were discussed in the multidisciplinary cancer board for treatment recommendations. Surgical resection, local ablation therapy, and TACE were employed as previously reported [23,24,25]. Patients were randomly assigned to derivation (*n* = 1898) and validation cohorts (*n* = 1896) at a 1:1 ratio for the construction and validation of the new model. This study was approved by the Institutional Review Board of Taipei Veterans General Hospital, and complies with the standards of the Declaration of Helsinki and current ethical guidelines.

### 2.2. Definition and Diagnosis

HCC was diagnosed according to the European Association of the Study of Liver (EASL) and American Association for the Study of Liver Diseases (AASLD) HCC practice guidelines [2,3]. Vascular invasion was defined as tumor invasion of portal veins or their branches, hepatic veins, or large vasculatures such as the inferior vena cava [26]. CT or MRI was performed to detect possible distant metastasis, as previously described [27,28]. Surgical resection, local ablation therapy, and liver transplantation were classified as curative treatments; TACE, systemic therapy, and best supportive care were defined as non-curative treatments.

### 2.3. Calculation of TBS

TBS was defined as the distance from the origin on a Cartesian plane that incorporated two variables: maximum tumor size, and number of liver lesions. The TBS was calculated by using the following equation: TBS^2^ = (maximum tumor diameter)^2^ + (number of tumors)^2^. TBS was divided into 3 groups—low (<3.36), medium (3.36 to 13.74), and high TBS (>13.74)—as described previously [12,13].

### 2.4. Calculation of ALBI Score 

The equation for ALBI score is as follows: ALBI score = (0.66 × log_10_ bilirubin (μmol/L) − (0.085 × albumin (g/L)). The cutoff values of ALBI grade 1/2 and grade 2/3 were −2.60 and −1.39, respectively [18].

### 2.5. Development and Validation of the New Prognostic Model 

The new prognostic model was constructed according to the following criteria: (1) the new method contains only clinically available parameters, and (2) it should be easy to use and calculate. The model was investigated in the derivation cohort with parameters including baseline characteristics, extent of tumor involvement, hepatic functional reserve, and performance status. Significant predictors in univariate survival analysis were evaluated by the multivariate Cox proportional hazards model to identify independent predictors. The derived predictors were employed to construct the new model in the derivation cohort. A new prediction score was proposed, giving ordinal scores (0, 1, and 2) to each of the independent predictors according to the calculated regression coefficients in the statistical model. This newly proposed TBS–ALBI system was established by adding each point of the predictors, and justified in the validation cohort according to differences in BCLC stage, treatment, and etiology of liver disease.

### 2.6. Statistics

We used IBM SPSS Statistics for Windows, version 21.0 (IBM Corp., Armonk, NY, USA), for statistical analysis. The comparison of continuous data and categorical data was carried out via Mann–Whitney U test and the chi-squared or Fisher’s exact test, respectively. Kaplan–Meier analysis was used to evaluate the survival differences in cancer patients. A multivariate Cox hazards model was applied to identify prognostic factors. The corrected Akaike information criteria (AICc) were obtained to reveal how staging systems were correlated with survival. The lower the AICc value, the more accurate and informative the model in terms of survival prediction [29]. A *p*-value < 0.05 was considered statistically significant.

## 3. Results

### 3.1. Baseline Characteristics

**Figure 1:**
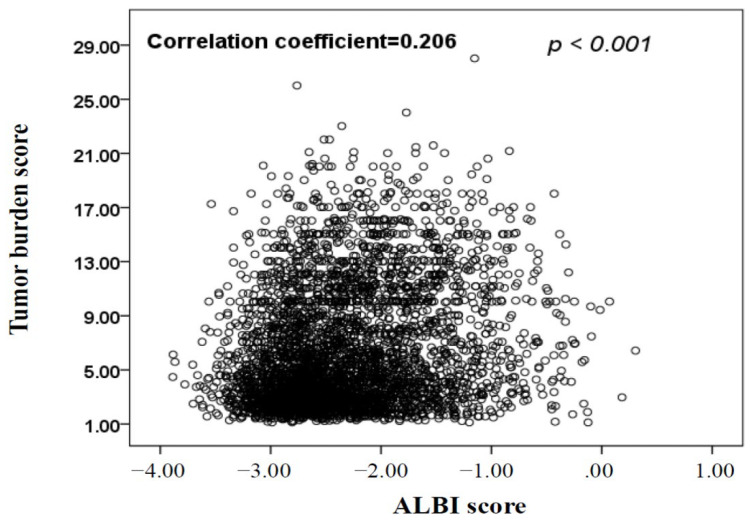
The correlation between tumor burden score (TBS) and albumin–bilirubin (ALBI) score. There was weak but significant correlation between TBS and ALBI score (correlation coefficient = 0.206, *p* < 0.001; *n* = 3794).

A prospective cohort of 3794 HCC patients was enrolled; their baseline characteristics are shown in Table 1. The mean age was 66 years, and most (76%) patients were male. HBV (40%) and HCV (22%) were the main etiological drivers of HCC. The median TBS was 5.0, and 31% and 61% of patients had low TBS and medium TBS, respectively. Vascular invasion or distant metastasis was found in 27% of patients, and 29% had serum α-fetoprotein (AFP) levels ≥ 400 ng/mL. CTP class A was noted in 73% of patients, and 38% and 52% of patients had ALBI grade 1 and grade 2, respectively. Fifty-two percent of patients received curative treatments. The distribution of patients according to different cancer staging systems is shown in Table 1. There were no significant baseline differences between the derivation and validation cohorts (all *p* > 0.05, Table 1). TBS was weakly yet significantly and positively correlated with ALBI score (correlation coefficient = 0.206, *p* < 0.001; Figure 1).

### 3.2. Development of the New TBS–ALBI Prognostic System

In univariate analysis, presence of ascites, serum AFP ≥ 400 ng/mL, vascular invasion or distant metastasis, performance status 2–4, TBS, and ALBI grade were significant variables associated with survival. In the multivariate Cox model, presence of ascites (HR: 1.343, 95% CI: 1.169–1.542, *p* < 0.001), AFP ≥ 400 ng/mL (HR: 1.523, 95% CI: 1.345–1.726, *p* < 0.001), vascular invasion or distant metastasis (HR: 2.462, 95% CI: 2.126–2.851, *p* < 0.001), performance status 2–4 (HR: 1.853, 95% CI: 1.612–2.129, *p* < 0.001), medium TBS (HR: 1.655, 95% CI: 1.446–1.893, *p* < 0.001), high TBS (HR: 2.395, 95% CI: 1.919–2.990, *p* < 0.001), ALBI grade 2 (HR: 1.879, 95% CI: 1.661–2.126, *p* < 0.001), and ALBI grade 3 (HR: 3.090, 95% CI: 2.540–3.761, *p* < 0.001) were independent predictors of increased mortality (Table 2). 

The TBS–ALBI system was developed based on the six predictors from the multivariate model, and the weight score of each variable was based on the predicted risk model. In this new TBS–ALBI system, two points are given for high TBS and ALBI grade 3, and one point is given for medium TBS, ALBI grade 2, presence of vascular invasion, presence of ascites, AFP ≥ 400 ng/mL, and performance status 2–4; the total score ranges from 0 to 8 points accordingly (Table 3).

### 3.3. Patient Survival in the Derivation Cohort Based on the TBS–ALBI System

**Figure 2:**
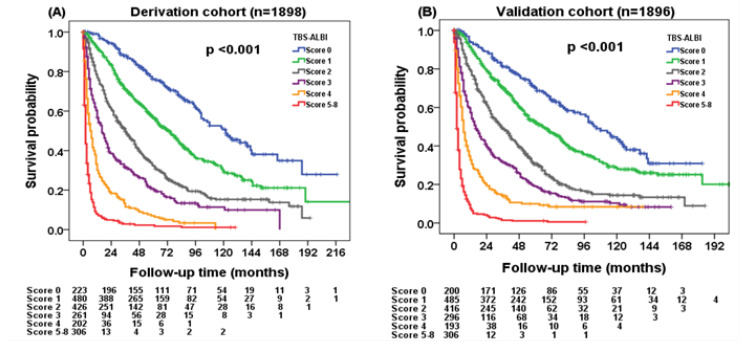
Kaplan–Meier analysis of the TBS- and ALBI-grade-based prognostic model (TBS–ALBI system) in the (**A**) derivation and (**B**) validation cohorts. There were significant survival differences in different TBS–ALBI score risk groups in the derivation cohort (*p* < 0.001; *n* = 1898). Patients with high TBS–ALBI scores consistently had decreased overall survival in the validation cohort (*p* < 0.001; *n* = 1896).

In this 7967 person-year study, the median overall survival was 125 (95% CI: 102–148) months, 70 (95% CI: 61–79) months, 36 (95% CI: 30–41) months, 16 (95% CI: 13–19) months, 5 (95% CI: 3.5–6.5) months, and 2 (95% CI: 1.7–2.3) months for TBS–ALBI scores of 0, 1, 2, 3, 4, and 5–8 points, respectively. The 1-, 3-, and 5-year survival rates were 99%, 85%, and 73% for TBS–ALBI score 0, 92%, 71%, and 65% for score 1, 76%, 50%, and 30% for score 2, 58%, 31%, and 22% for score 3, 32%, 12%, and 6% for score 4, and 9%, 3%, and 3% for scores of 5–8, respectively. Patients with higher TBS–ALBI scores had decreased survival compared with lower TBS–ALBI scores in the derivation cohort (Figure 2A, *p* < 0.001).

### 3.4. Validation of the TBS–ALBI System

Significant survival differences were found in different TBS–ALBI score groups of the validation cohort (Figure 2B, *p* < 0.001). The 1-, 3-, and 5-year survival rates were 94%, 81%, and 68% for TBS–ALBI score 0, 90%, 70%, and 52% for score 1, 76%, 47%, and 29% for score 2, 56%, 30%, and 18% for score 3, 32%, 14%, and 11% for score 4, and 8%, 2%, and 2% for scores of 5–8, respectively.

The prognostic performance of the TBS–ALBI system was compared with other staging systems, including BCLC, HKLC, TIS, JIS, CLIP, Okuda, Tokyo, and TNM (Table 4). The TBS–ALBI system had the lowest AICc and the highest homogeneity, indicating better prognostic performance than other systems. In subgroup analysis stratified by curative (*n* = 902) and non-curative treatments (*n* = 994), the TBS–ALBI system still outperformed other staging systems. The TBS–ALBI system was consistently a better prognostic model when stratified by the etiology of HBV (*n* = 776) and HCV (*n* = 374) groups.

### 3.5. Using the TBS–ALBI System to Differentiate Survival in Different Risk Groups

**Figure 3:**
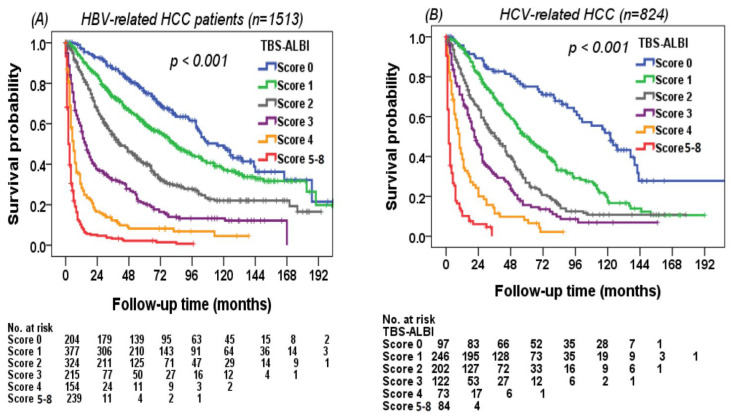
Survival distribution according to the TBS–ALBI system in (**A**) HBV-related HCC and (**B**) HCV-related HCC. Significant survival differences were found between different TBS–ALBI score groups in HBV-related (*p* < 0.001; *n* = 1513) and HCV-related HCC (*p* < 0.001, *n* = 824).

**Figure 4:**
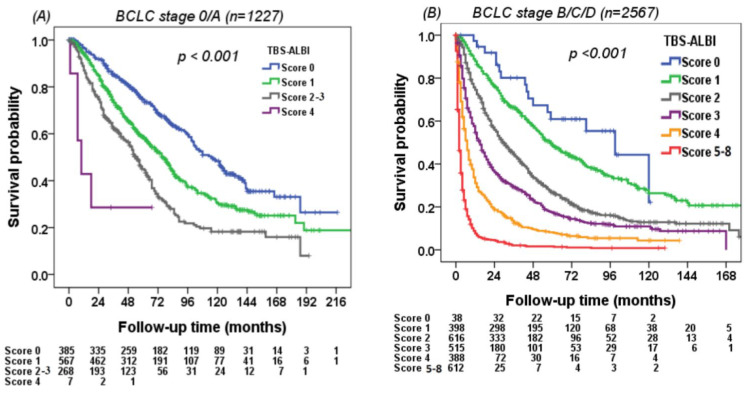
Survival distribution according to the TBS–ALBI system in (**A**) BCLC stage 0/A patients and (**B**) BCLC stage B–D patients. Patients with lower TBS–ALBI scores had better overall survival compared with those with higher scores in BCLC stage 0/A (*p* < 0.001; *n* = 1227) and BCLC stages B–D (*p* < 0.001, *n* = 2567).

**Figure 5:**
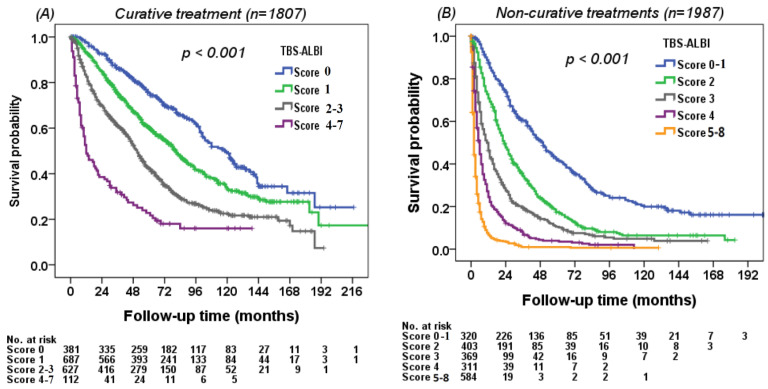
Survival distribution according to the TBS–ALBI system in patients receiving (**A**) curative and (**B**) non-curative treatments. Patients with high TBS–ALBI scores had increased risk of mortality compared with those with lower scores in patients receiving curative (*p* < 0.001; *n* = 1807) and non-curative treatments (*p* < 0.001; *n* = 1987).

The discriminatory ability of survival for the TBS–ALBI system was evaluated in all HCC patients. The TBS–ALBI system can differentiate survival status well in either HBV-related or HCV-related HCC (*p* < 0.001, Figure 3A,B). The survival difference was consistently identified in patients stratified by BCLC stage 0/A (*n* = 1227) and stage B/C/D patients (*n* = 2567) (*p* < 0.001, Figure 4A,B). Patients with lower TBS–ALBI scores had better overall survival compared with higher TBS–ALBI scores stratified by curative (*n* = 1807) and non-curative treatments (*n* = 1987) (*p* < 0.001, Figure 5A,B).

## 4. Discussion

Staging systems provide crucial information in prognostic prediction for cancer patients. Quite a few staging systems have been proposed for outcome prediction in HCC, but the optimal model remains highly debatable. The BCLC staging system is currently the recommended system for HCC, and is included in the practice guidelines. However, a major drawback of the BCLC is that the outcomes are highly variable even for patients within the same stage [13,30]. In this study, we proposed and validated a new prognostic model—the TBS–ALBI system—for HCC. The TBS–ALBI system offers superior prognostic performance in comparison with other staging systems. In addition, its prognostic ability is stably consistent in patients with viral HCC. Notably, the TBS–ALBI score can discriminate overall survival well in patients with different BCLC stages and treatment strategies. Thus, the TBS–ALBI system is a very informative prognostic tool for HCC patients with different clinical characteristics. 

Tumor burden, severity of liver dysfunction, and performance status are known important prognostic predictors for HCC. Typically, the size and number of tumor nodules are used to assess tumor burden, and have been included in many staging systems. These two variables are binary in nature, and could make it difficult to clearly differentiate the outcomes of HCC patients with variable tumor size and numbers. For example, the prognosis is quite different in patients with a single large HCC of 6 cm compared to those with three nodules of 4 cm, 3 cm, and 2 cm in diameter. Thus, shifting from dichotomous to continuous variables of tumor size and number may enhance the prognostic ability. By using the Pythagorean theorem, TBS considers the collective impact of tumor diameter and number of tumors, encompassing the magnitude of tumor burden [12,15]. As such, TBS is a single and continuous variable to indicate tumor burden in HCC, as opposed to dichotomous variables such as the Milan criteria and Up-to-7 criteria. In addition, TBS is easy to calculate, requiring only maximal tumor diameter and number of nodules, as opposed to other calculation methods that require the diameter and number of all tumors. We found that patients with high TBS had a higher mortality risk than those with low and medium TBS in the multivariate Cox model. Consistent with previous studies [13,14,15,31,32], our study confirms that TBS is a feasible prognostic marker to assess tumor burden in HCC.

The management of HCC is associated with underlying liver dysfunction. The CTP classification and MELD score were designed for HCC patients with cirrhosis. However, these two models have potential limitations, because ~20% of HCCs arise from non-cirrhotic livers. In this regard, the ALBI grade is a more objective marker of liver dysfunction, and can discriminate patient survival well, with high predictive accuracy [18,19,20,21,22]. In this study, ALBI grade 1 patients clearly had better survival compared with ALBI grade 2 and 3 patients in the multivariate Cox model. This finding consistently suggests that ALBI grade is a reliable prognostic tool to evaluate hepatic reserve in HCC.

Vascular invasion and distant metastasis typically indicate advanced cancer stages, and are often associated with large tumor burden in HCC [27,33]. In this study, patients with vascular invasion or metastasis had 2.3-fold increased risk of mortality compared with those without these features. Alternatively, ascites is a classical hallmark of portal hypertension [34]. The presence of ascites in HCC patients indicates not only poor hepatic functional reserve, but also aggressive tumor behavior [35]. In multivariate analysis, the presence of ascites was associated with a 34% increased risk of mortality compared with patients without ascites. Consistently with previous studies [35,36], we can confirm that ascites is an indispensable prognostic predictor for HCC.

AFP is a widely used biomarker for HCC in both diagnosis and prognostic prediction [37]. AFP is intimately associated with cell proliferation and cancer progression [38,39]. AFP also tightly coexists with large tumor burden, and predicts decreased survival in HCC patients [37]. Our data further suggest that AFP is an important parameter in prognostic prediction for HCC. Another meaningful finding in this study is our confirmation that performance status is a strong surrogate to indicate patient survival. Patients with a suboptimal performance status had a 1.8-fold increased risk of mortality compared with those with good performance status. Taken together, TBS, ALBI grade, ascites, vascular invasion or distant metastasis, AFP, and performance status are all crucial prognostic determinants for HCC. By using these six predictors, we developed and validated the novel TBS–ALBI system to predict long-term outcomes in HCC patients.

The TBS–ALBI system has several strengths. Firstly, TBS calculates tumor burden in a continuous fashion, and can stratify different risk groups to discriminate outcomes in HCC patients. Secondly, the ALBI grade is a simple and objective method to indicate hepatic functional reserve, with good prognostic performance in HCC patients, since most HCCs have mild fibrosis or cirrhosis at the time of diagnosis. Thirdly, the TBS–ALBI system is derived from clinical and objective laboratory variables, and is easy to calculate, without the need for complex computing to assess prognosis. Lastly, the TBS–ALBI system offers superior prognostic performance compared with other staging systems for HCC. Thus, the TBS–ALBI system could be a novel tool for making clinical decisions for patients with HCC.

There are nevertheless a few limitations to the present study. Firstly, the TBS–ALBI system was developed from a single center in Taiwan. External validation from different geographic regions is required in order to validate our results. Secondly, although TBS is a feasible marker to indicate tumor burden, the tumor diameter and number of nodules have the same prognostic statistical weight, which may require adjustment. Lastly, anticancer treatments are primarily decided based on shared decision making; as such, some patients may not strictly adhere to the BCLC recommendations.

## 5. Conclusions

The TBS–ALBI system, derived from six clinical predictors, is a novel, simple, and user-friendly prognostic model to evaluate HCC; its prognostic performance is better than that of other currently used staging systems. The predictive ability of the TBS–ALBI system remains consistently stable across different BCLC stages, treatments, and viral etiologies for outcome evaluation. External validation from different study groups is required in order to further validate our results.

## Figures and Tables

**Table 1:** Baseline demographics (*n* = 3794).

Variables	All Patients	Derivation Cohort (*n* = 1898)	Validation Cohort (*n* = 1896)	*p*
Age (years, mean ± SD)	66 ± 13	64 ± 13	65 ± 13	0.715
Male, *n* (%)	2895 (76)	1450 (76)	1445 (75)	0.909
Etiologies of liver disease				0.894
HBV, *n* (%)	1513 (40)	737 (39)	776 (41)	
HCV, *n* (%)	824 (22)	450 (24)	374 (20)	
HBV + HCV, *n* (%)	135 (3)	61 (3)	74 (4)	
Others, *n* (%)	1322 (35)	650 (34)	672 (35)	
Performance status (0/1/2/3–4), *n* (%)	2226/780/431/357 (59/21/11/9)	1119/376/218/185 (59/20/12/9)	1107/404/213/172 (59/21/11/9)	0.758
Diabetes mellitus, *n* (%)	972 (26)	459(25)	503 (27)	0.206
Tumor nodules (single/multiple), (%)	2437/1357 (64/36)	1227/671 (65/35)	1210/686 (64/36)	0.611
Maximal tumor diameter ≥5 cm, *n* (%)	1668 (44)	838 (44)	830 (44)	0.819
Tumor burden score (mean ± SD)	5.0 ± 4.4	6.5 ± 4.3	6.5 ± 4.4	0.550
Tumor burden score (low/medium/high)	1160/2299/335 (31/61/8)	578/1157/163 (30/61/9)	582/1142/172 (31/60/9)	0.900
Vascular invasion or distant metastasis, *n* (%)	1038 (27)	494 (26)	544 (28)	0.069
Serum AFP ≥ 400 ng/mL, *n* (%)	1117 (29)	546 (29)	571 (30)	0.373
Ascites, *n* (%)	861 (23)	432 (23)	429 (23)	0.938
Laboratory values (mean ± SD)				
Albumin (g/L)	37 ± 6	36 ± 6	36 ± 6	0.600
Total bilirubin (μmol/L)	15 ± 48	26 ± 51	26 ± 44	0.685
Platelets (1000/μL)	153 ± 96	170 ± 95	170 ± 97	0.938
INR of prothrombin time	1.06 ± 0.2	1.1 ± 0.3	1.1 ± 0.2	0.187
Creatinine (mg/dL)	1.0 ± 1.0	1.2 ± 1.0	1.2 ± 1.0	0.944
CTP class (A/B/C)	2787/831/176 (73/22/5)	1480/386/104 (74/20/6)	1379/445/72 (72/24/4)	0.006
CTP score, mean ± SD	5.0 ± 1.5	6.0 ± 1.5	6.0 ± 1.5	0.644
ALBI grade (1/2/3), *n* (%)	1444/1970/380 (38/52/10)	745/943/210 (39/50/11)	699/1027/170 (37/54/9)	0.899
ALBI score, mean ± SD	−2.40 ± 0.65	−2.29 ± 0.66	−2.30 ± 0.63	0.810
Tumor staging, (%)				
BCLC stage (0/A/B/C/D)	8/24/17/40/11	8/24/18/38/11	8/25/16/41/11	0.754
HKLC (I/II/III/IV/V)	32/27/10/8/22	32/27/10/8/23	32/27/10/9/22	0.885
TIS (0/1/2/3/4/5/6)	36/22/21/12/12/11/6/1	37/21/13/11/12/5/1	36/22/12/12/11/6/1	0.378
JIS (0/1/2/3/4/5)	9/33/30/17/9/2	9/33/30/17/9/2	9/33/30/17/9/1	0.827
CLIP (0/1/2/3/4/5/6)	32/26/15/12/9/5/1	33/26/15/12/9/4/1	31/27/15/11/10/5/1	0.346
Okuda (1/2/3)	53/38/9	53/38/9	53/39/8	0.616
Tokyo (0/1/2/3/4/5/6/7/8)	6/22/26/19/12/8/4/2/1	6/23/25/19/12/8/4/2/1	6/21/27/19/13/8/3/2/1	0.833
TNM (I/II/III/IV)	33/25/36/6	34/24/36/6	33/24/36/6	0.681
Treatments, *n* (%)				0.775
Surgical resection	1107 (29)	569 (30)	538 (28)	
Local ablation therapy	680 (18)	327 (17)	353 (19)	
TACE	1034 (27)	521 (27)	513 (27)	
Liver transplantation	20 (1)	9 (1)	11 (1)	
Targeted therapy	303 (8)	154 (8)	149 (8)	
Others	650 (17)	318 (17)	332 (17)	

ALBI: albumin–bilirubin; AFP: alpha-fetoprotein; BCLC: Barcelona Clinic Liver Cancer; CTP: Child–Turcotte–Pugh score; HBV: hepatitis B virus; HCV: hepatitis C virus; SD: standard deviation; TACE, transarterial chemoembolization.

**Table 2:** Univariate and multivariate analysis of overall survival in HCC patients in the derivation cohort (*n* = 1898).

Variables	Number	Univariate Analysis			Multivariate Analysis		
		3-Year Survival (%)	5-Year Survival (%)	*p*	Hazard Ratio	95% CI	*p*
Age (<55/≥55 years)	955/943	44/47	37/34	0.936			
Sex (male/female)	2895/899	64/73	45/52	0.004			
HBV (negative/positive)	905/993	45/48	31/38	0.041			
HCV (negative/positive)	1311/587	46/48	35/35	0.462			
Platelet (<150,000/≥150,000/μL)	986/912	43/50	17/13	0.034			
Ascites (absent/present)	1466/432	55/18	41/13	<0.001	1.343	1.169–1.542	<0.001
Serum AFP (<400/≥400 ng/mL)	1352/546	56/22	43/15	<0.001	1.523	1.345–1.726	<0.001
Vascular invasion or distant metastasis (no/yes)	1404/494	57/9	43/6	<0.001	2.312	2.010–2.658	<0.001
Diabetes mellitus (no/yes)	1429/469	48/43	36/31	0.025			
Performance status 0–1/2–4	1119/709	54/18	42/9	<0.001	1.853	1.612–2.129	<0.001
Tumor burden score							
Low	578	71	56				
Medium	1157	39	28	<0.001	1.655	1.446–1.893	<0.001
High	163	9	6	<0.001	2.395	1.919–2.990	<0.001
ALBI							
Grade 1	745	68	55				
Grade 2	943	37	25	<0.001	1.879	1.661–2.126	<0.001
Grade 3	210	11	7	<0.001	3.090	2.540–3.761	<0.001

HBV: hepatitis B virus; HCV: hepatitis C virus; AFP: α-fetoprotein; ALBI: albumin-bilirubin.

**Table 3:** The TBS–ALBI system.

Prognostic Factors	Score		
	0	1	2
Tumor burden score	Low	Medium	High
ALBI grade	1	2	3
Vascular invasion or distant metastasis	Absent	Present	
Ascites	Absent	Present	
Serum AFP (ng/mL)	<400	≥400	
Performance status	0–1	2–4	

**Table 4:** Prognostic performance of different staging systems in the validation cohort.

Models	Homogeneity (Wald χ^2^)	Corrected Akaike Information Criterion
Validation cohort (*n* = 1896)		
BCLC	477.462	18,747.588
HKLC	543.712	18,681.338
TIS	676.704	18,544.279
JIS	503.527	18,721.523
CLIP	756.433	18,468.617
Okuda	466.310	18,758.439
Tokyo	517.941	18,707.108
TNM	294.309	18,930.740
TBS–ALBI	871.542	18,353.503
Curative treatment (*n* = 902)		
BCLC	35.453	6429.229
HKLC	36.586	6428.089
TIS	35.420	6427.088
JIS	39.044	6425.638
CLIP	51.544	6413.138
Okuda	30.110	6434.517
Tokyo	36.884	6427.798
TNM	12.795	6451.887
TBS–ALBI	76.604	6388.087
Non-curative treatment (*n* = 994)		
BCLC	250.074	10,420.458
HKLC	268.381	10,402.051
TIS	380.781	10,289.651
JIS	241.444	10,428.988
CLIP	372.307	10,298.125
Okuda	205.407	10,465.025
Tokyo	198.510	10,471.922
TNM	162.579	10,507.853
TBS–ALBI	432.154	10,238.278
HBV-related HCC (*n* = 776)		
BCLC	201.496	6260.271
HKLC	225.089	6235.670
TIS	257.214	6202.857
JIS	204.453	6257.314
CLIP	278.184	6183.584
Okuda	181.108	6280.659
Tokyo	203.245	6258.522
TNM	130.840	6330.928
TBS–ALBI	359.468	6102.300
HCV-related HCC (*n* = 374)		
BCLC	115.183	2894.521
HKLC	135.930	2873.744
TIS	144.346	2863.157
JIS	105.382	2904.322
CLIP	185.719	2823.985
Okuda	85.960	2923.744
Tokyo	97.339	2682.659
TNM	47.638	2962.066
TBS-ALB	189.422	2820.261

## Data Availability

The data presented in this study are available on request from the corresponding author. The data are not publicly available due to ethical concerns.
